# A new model for fatty acid hydroxylase-associated neurodegeneration reveals mitochondrial and autophagy abnormalities

**DOI:** 10.3389/fcell.2022.1000553

**Published:** 2022-12-14

**Authors:** Frida Mandik, Yuliia Kanana, Jost Rody, Sophie Misera, Bernd Wilken, Björn-Hergen Laabs von Holt, Christine Klein, Melissa Vos

**Affiliations:** ^1^ Institute of Neurogenetics, University of Luebeck, UKSH, Luebeck, Germany; ^2^ Department of Neuropediatrics, Klinikum Kassel, Kassel, Germany; ^3^ Institut für Medizinische Biometrie und Statistik, University of Luebeck, Luebeck, Germany

**Keywords:** fatty acid hydroxylase-associated neurodegeneration, *Drosophila melanogaster*, FA2H, autophagy, mitochondria

## Abstract

Fatty acid hydroxylase-associated neurodegeneration (FAHN) is a rare disease that exhibits brain modifications and motor dysfunctions in early childhood. The condition is caused by a homozygous or compound heterozygous mutation in *fatty acid 2 hydroxylase* (*FA2H*), whose encoded protein synthesizes 2-hydroxysphingolipids and 2-hydroxyglycosphingolipids and is therefore involved in sphingolipid metabolism. A few FAHN model organisms have already been established and give the first insight into symptomatic effects. However, they fail to establish the underlying cellular mechanism of FAHN so far. *Drosophila* is an excellent model for many neurodegenerative disorders; hence, here, we have characterized and validated the first FAHN *Drosophila* model. The investigation of loss of dfa2h lines revealed behavioral abnormalities, including motor impairment and flying disability, in addition to a shortened lifespan. Furthermore, alterations in mitochondrial dynamics, and autophagy were identified. Analyses of patient-derived fibroblasts, and rescue experiments with human FA2H, indicated that these defects are evolutionarily conserved. We thus present a FAHN *Drosophila* model organism that provides new insights into the cellular mechanism of FAHN.

## 1 Introduction

Fatty Acid Hydroxylase-associated Neurodegeneration (FAHN) is a neurodegenerative disease presenting with symptoms in early childhood. The most common clinical findings are spasticity, cognitive impairment, ataxia, and dystonia (Levi and Tiranti, 2019). FAHN is part of a heterogeneous group of neurodegenerative diseases called Neurodegeneration with brain iron accumulation (NBIA), characterized by iron depositions in the brain. These can be detected by magnetic resonance imaging (MRI) and are typically located in the globus pallidus and substantia nigra (Arber et al., 2016). MRI images of FAHN patients show features described by the acronym “WHAT”: White matter changes, hypointensity of the globus pallidus resulting from iron accumulation, pontocerebellar **a**trophy, and a **t**hinned corpus callosum. Examination of a large group of FAHN patients showed that at least three of the four MRI findings occur in FAHN patients. Brain iron deposition was observed in 77% of patients (Rattay et al., 2019).

While the underlying mechanisms remain elusive, the genetic defect was identified, namely homozygous or compound heterozygous mutations in *fatty acid 2 hydroxylase* (*FA2H*). It encodes the eponymous protein, FA2H, localized in the endoplasmic reticulum and expressed mainly in the *epidermis*, brain, and colon (Alderson et al., 2004; Uchida et al., 2007). It is involved in the synthesis of 2-hydroxysphingolipids and 2-hydroxyglycosphingolipids (Eckhardt et al., 2005). Few disease models have currently been established and provide first insights into the symptomatic effects of *FA2H* mutations. In mouse models, loss of FA2H leads to degradation of axons and myelin and an altered myelin protein expression (Zöller et al., 2008; Potter et al., 2011; Hardt et al., 2020). In addition, deficits in spatial learning and memory were identified in mutant mice (Potter et al., 2011). Analysis of a *C. elegans* FAHN model showed impaired growth, a shorter lifespan, and inhibited lipid droplet formation (Li et al., 2018).

Nevertheless, current studies in animal models have not yet established the cellular effects of loss of FA2H. In addition, one animal model usually does not replicate all disease signs and symptoms (Nagoshi, 2018), which increases the need for additional animal models. A suitable animal model for the analysis of neurogenerative diseases and their underlying cellular mechanisms is *Drosophila melanogaster* (Bier, 2005; Nagoshi, 2018; Bolus et al., 2020; Vos and Klein, 2021). Although it is a relatively simple organism, it features a complex neurological system, has orthologs of many disease-causing genes, and numerous tools are available for genetic manipulation and functional analysis (Reiter et al., 2001; Bier, 2005; Yamamoto and Seto, 2014; Nagoshi, 2018). Furthermore, established *Drosophila* models for other neurodegenerative diseases such as Parkinson’s disease (PD) or other NBIAs showed behavioral abnormalities similar to those observed in humans, and they contributed to deciphering underlying cellular disease mechanisms (Park et al., 2006; Wu et al., 2009; Kinghorn et al., 2015; Julienne et al., 2017).

In this manuscript, we present a loss of dfa2h *Drosophila* model that exhibits clinical signs of FAHN, such as reduced lifespan and impaired movement. In addition, these flies demonstrated mitochondrial changes and altered autophagy, giving first insights into the cellular dysfunction due to loss of dfa2h. Furthermore, these cellular processes are evolutionarily conserved and of translational relevance, as we found similar discrepancies in patient-derived fibroblasts. Furthermore, we showed rescue of the loss of dfa2h phenotypes by overexpression of human FA2H. Thus, the model organism established here provides an excellent tool to investigate FA2H function further and allows us to obtain novel insights into FAHN.

## 2 Methods

### 2.1 Fly genetics and handling

The homozygous transposable mutant fly stocks *W^1118^*;*PBac{RB}fa2h^e00486^* (*dfa2h^1^*) and *W^1118^*;*PBac{WH}fa2h^f01498^* (*dfa2h^2^*), wild type control *W* ^*1118*^ (*control* ^*WT*^) and W^1118^;DaGal4 were purchased from the Bloomington *Drosophila* Stock Center (Indiana, United States). The heterozygous controls *dfa2h* ^*1*^ */+* and *dfa2h* ^*2*^ */+* were generated by crossing the mutant lines with *W* ^*1118*^. The results for the heterozygous controls were pooled (*control* ^*het*^). The compound heterozygous line *dfa2h* ^*1*^ */dfa2h* ^*2*^ was generated by crossing the mutant lines *dfa2h* ^*1*^ and *dfa2h* ^*2*^ with each other. The flies were kept at 25°C and fed with standard molasse food. Male flies were used for the experiments.

### 2.2 cDNA analysis

RNA was isolated using the Monarch Total RNA Miniprep Kit (T2010S) from New England Biolabs. For RNA isolation from *Drosophila,* 20 flies were homogenized in 250 µL 1x Protection Buffer following the “Tissue or Leukocytes” protocol. The RNA from 500.000 fibroblasts cultivated overnight at 37°C was isolated according to the “Cultured Mammalian Cells” protocol. A DNase I treatment was performed, and the RNA was eluted in 50 µL nuclease-free water. cDNA was generated using the Maxima First Strand cDNA Synthesis Kit with dsDNase from Thermo Scientific (K1672). As a noRT control, water was used instead of the maxima enzyme mix. Quantitative real-time PCR was performed using the Maxima SYBR Green/Fluorescein qPCR Master Mix from Thermo Scientific (K0243) and the LightCycler96 from Roche. *Rpl32*, *eEF1*α2, and *Act5c* were used as reference genes for fly cDNA. The results of the different references were merged, and the mean value was calculated. Primers are listed in the Supplementary Table S1.

### 2.3 Survival assay

The survival of flies was tracked by dividing flies into groups of ten. Their survival was documented every 2–3 days. Every seven days, the flies were transferred to fresh food.

### 2.4 Behavioral experiments

To determine the number of flies with “held-up” wings, flying ability, and motor disturbances, populations of four to five flies were formed. The number of flies with raised wings was counted in one-week-old flies and presented as percentages relative to the total number of tested flies. Flying ability and motor disturbances were tested in one-week-old and three-week-old flies by transferring the flies to an empty vial and stimulating the flies *via* light tapping. For flying ability, the number of flies capable of flying was determined and for motor disturbances, the number of flies with twitching, shaking, or uncoordinated flying was monitored.

### 2.5 Patient-derived Fibroblasts

Patient-derived skin fibroblasts carry a compound heterozygous mutation (c. [704G>A]; [ 859T>C] p. [R235H]; [C287R]) and were previously described (Rattay et al., 2019). Fibroblasts from two healthy controls were used, matching sex and ethnicity. The results from these controls were combined, and the mean value was calculated. All fibroblasts were maintained in Dulbecco’s modified eagle’s medium from Gibco (11995-065) supplemented with 10% fetal bovine serum from Gibco (26140087).

### 2.6 Immunolabeling

Immunolabeling was performed according to standard immunohistochemistry protocols (Vos et al., 2021). In brief, third instar larvae were dissected and fixed with 4% paraformaldehyde in PBS for 20 min and permeabilized with 0.4% Triton X-100 in PBS. Primary antibodies anti-ATP5A (15H4C4) (1:200; ab14748) and anti-GABARAP (dLC3) (1:167; ab109364) from Abcam and secondary antibodies, goat anti-Rabbit Alexa Fluor 488 (1:250; A11034), and goat anti-Mouse Alexa Fluor 594 (1:250; A11032) from Invitrogen were used. Antifade mounting medium with DAPI from Vectashield (H1200) was used to mount the larvae. Fibroblasts were fixed for 15 min in 4% paraformaldehyde. Primary antibodies anti-GRP75 from Abcam (1:1000; ab53098) and anti-LC3A/B from Cell Signaling (1:150; 4108), and secondary antibodies, goat anti-Rabbit Alexa Fluor 488 (1:200; A11034), and goat anti-Rabbit Alexa Fluor 594 (1:400; 11012) from Invitrogen were used. DAPI-Fluoromount-G^®^ from SouthernBiotech (0100-01) was used for mounting. The LSM70 confocal microscope with 63x NA 1.4 oil lens was used to image the cells and larval muscle sections, and ImageJ was used to analyze the fluorescent signals. The mitochondrial morphology macro from Ruben K. Dagda automated mitochondria field evaluation (Dagda et al., 2009). The interconnectivity was studied by the ratio of the area/perimeter (Wiemerslage and Lee, 2016). Particles with a minimal size of 0.035 µm^2^ in flies and 0.4 µm^2^ in fibroblasts were included in the calculation. The number of LC3 accumulations was calculated using the automated “Triangle” threshold and the “Analyze Particles” plugin. Here, particles with a minimal size of 0.1 µm^2^ in flies and 0.4 µm^2^ in fibroblasts were included in the calculation.

### 2.7 Western blot analysis

Protein was extracted from three-week-old flies and fibroblasts using RIPA extraction buffer (25 mM TRIS-HCl pH 7.6, 20°C, 150 mM NaCl, 1% (V/V) NP-40, 1% (V/V) DOC, 0.1% (V/V) SDS, cOmplete^tm^ mini protease inhibitor cocktail and PhosStop^tm^ from Roche). The gels were blotted on nitrocellulose membranes. As primary antibodies for fly samples, we used anti-GABARAP (dLC3) from Abcam (1:600; ab109364), anti-MFN2 from antibodies online (1:1000; ABIN4916092), and anti-Drp1 from Cell Signaling (1:2000; 8570). As primary antibodies for fibroblast samples, we used anti-LC3A/B from Cell Signaling (1:1000; 4108), anti-MFN2 from Abcam (1:1000; ab56889), and anti-DRP1 from Invitrogen (1:1000; PA1-16987). Anti-ß-Actin from Abcam (1:400000; ab8224) was used for both organisms as a loading control. As secondary antibodies, we used anti-mouse lgG HRP-linked from Cell Signaling (1:5000; 7076), and anti-rabbit lgG HRP-linked from Cell Signaling (1:5000; 7074) for both fly and fibroblast protein.

### 2.8 Hydrogen peroxide measurement

Hydrogen peroxide (H_2_O_2_) assay kit from Abcam (ab102500) was used following the “fluorometric” protocol to estimate H_2_O_2_ level. Ten three-week-old flies without heads were homogenized in 70 µL assay buffer.

### 2.9 Rescue experiment

The rescue experiments were performed by overexpressing human FA2H in dfa2h-deficient flies. Human FA2H cDNA was generated from human fibroblasts as described in the method section “cDNA analysis”. The cDNA was inserted into the overexpression vector “pUAST-attB” (Bischof et al., 2007) using the “NEBuilder Hifi DNA assembly cloning kit” following the manufacturer’s protocol. To generate cDNA with overhangs complementary to the vector, a PCR was performed using the Taq CORE Kit 10 from MP Biomedicals (11EPTQK101-CF) and the HiFi primers listed in Supplementary Table S1. The vector was linearized using the restriction enzymes KpnI-HF (R3142) and EagI-HF (R3505) from New England Biolabs according to the manufacturer’s protocol by incubation for 1 h at 37°C. The DNA gel extraction was performed using the QIAquick gel extraction kit from QIAGEN (28706X4), eluting in 30 µL EB-Buffer. To generate flies carrying this construct, the vector was microinjected in the line *y* ^*1*^ *w* ^*1118*^ *;PBac{y + -attP-3B}VK00033* (Bloomington *Drosophila* Stock Center stock number 9750). BestGene injected the embryo’s with the construct and selected the flies that carried the construct. The UAS promoter that is driving the ubiquitous overexpression is activated by DaGal4 (DG4) expression. As a control *dfa2h* ^*1*^ */+;UAS-FA2H/DG4,* and as a rescue line *dfa2h* ^*1*^ */dfa2h* ^*2*^ *;UAS-FA2H/DG4* were used. Values were normalized to their respective control (*control* ^*het*^ for *dfa2h* ^*1*^ */dfa2h* ^*2*^ *and dfa2h* ^*1*^ */+;UAS-FA2H/DG4* for *dfa2h* ^*1*^ */dfa2h* ^*2*^ *;UAS-FA2H/DG4).* We included the basic *control* ^*het*^ flies in the graphs to evaluate the rescue level (partial or full). For the analysis of the behavioral differences, three-week-old flies were used, except for the “held-up” wing observation, where one-week-old flies were used as this phenotype is age-independent.

### 2.10 Statistics

For all the experiments with a sample size >7, nonparametric analyses were performed using GraphPad Prism 8.4.3. For statistical comparison of more than two groups, we performed Kruskal-Wallis-Test with a significance level of *p* < 0.05. To further interpret differences between single groups, pairwise comparisons were performed using the Mann-Whitney-U-Test, with a significance level of *p* < 0.05 in experiments with two groups. The Bonferroni correction was used to account for multiple testing in experiments with more than two groups. This calculation adjusted the significance level depending on the number of tests made. Three tests were applied in experiments with three groups, resulting in an alpha of 0.05/3 = 0.017. With four groups, six tests were used, resulting in an alpha of 0.05/6 = 0.008, and for five groups, ten tests were applied, resulting in an alpha of 0.05/10 = 0.005. All pairwise tests with *p* < 0.05, *p* < 0.017, *p* < 0.008, and *p* < 0.005, respectively, were indicated with asterisks in the figure. We did group and pairwise curve comparisons using the Log-rank (Mantel-Cox) Test for statistical evaluation of the monitored survival.

## 3 Results

### 3.1 *Drosophila dfa2h*-mutant lines present with a reduced lifespan

**FIGURE 1:**
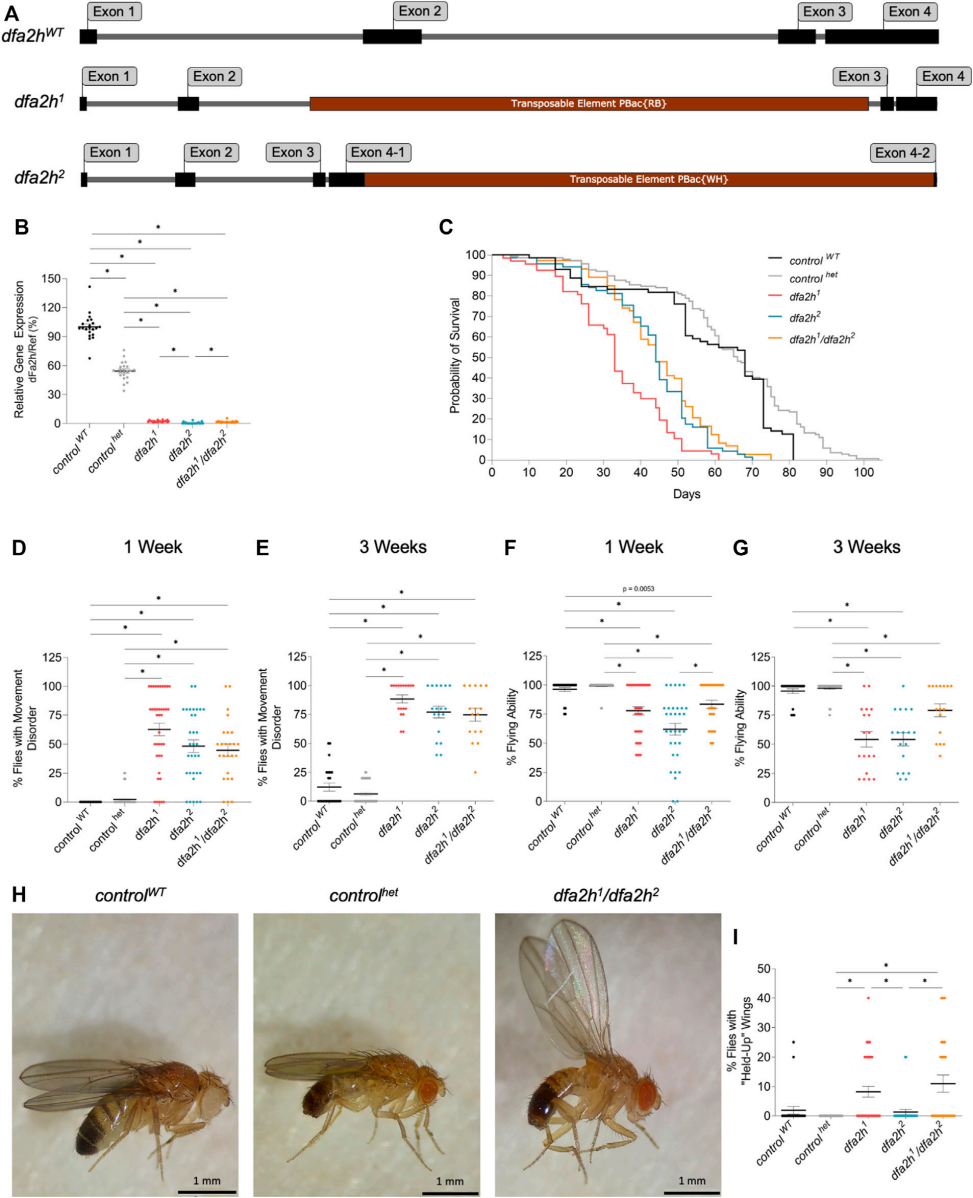
Transposable element insertion leads to reduced *dfa2h* cDNA level, causing reduced lifespan and motor disabilities. **(A)** Schematic image of the gDNA of *dfa2h* ^*WT*^ and the transposable element insertion lines *dfa2h* ^*1*^ and *dfa2h* ^*2*^. **(B)** Relative *dfa2h* level normalized to the combined data of the reference genes *Act5c*, *Rpl32,* and *eEF1α2* expressed as percentages relative to *control* ^*WT*^ that was set to 100%. The dots represent the single data points, and the lines show mean ± SEM (n = 21). **(C)** Survival monitoring of *dfa2h* mutant flies, *control* ^*het*^ and *control* ^*WT*^ (n = 67-137). Group curve comparisons were performed using log-Rank (Mantel-Cox) Test with a significance level of *p* < 0.05 (*). We performed a pairwise curve comparison using the log-Rank (Mantel-Cox) Test for better result interpretation. All pairwise comparisons, except the comparison of *dfa2h* ^*2*^ vs. *dfa2h* ^*1*^ */dfa2h* ^*2*^, were significant. **(D)** One-week-old and **(E)** three-week-old homozygous and compound heterozygous *dfa2h* flies display movement disabilities compared to control flies. **(F)** One-week-old and **(G)** three-week-old *dfa2h*-mutant flies show difficulties in flying capacity compared to control flies. **(H)** “Held-up” wing phenotype of *dfa2h* ^*1*^ */dfa2h* ^*2*^ compared to normal wing position in controls. **(I)** Evaluation of flies with “held-up” wing position displayed as the ratio of flies with “held-up” wing phenotype/total amount of flies tested. For the experiments **(B,D–G,I)**, the dots represent single data points as percentages relative to the number of flies observed per population, and the lines show mean ± SEM (n_one-week_ = 24–52; n_three-weeks_ = 16–52). The Kruskal–Wallis test was performed for group comparison with a significance level of *p* < 0.05 **(B,D–G,I)**.

Most disease-causing mutations in *FA2H* are missense or truncated mutations leading to a loss-of-function of FA2H (Darley et al., 1969; Edvardson et al., 2008; Rattay et al., 2019). In *Drosophila melanogaster*, there is a gene ortholog, *dfa2h,* that consists of four exons and entails a coding region that spans 1068 bp (Figure 1A). Several homozygous *dfa2h*-mutant lines containing a transposable element inserted in different regions of the gene exist. The first mutant fly line used in this paper is *dfa2h* ^*1*^, which has an insertion located centered in the gene between exon 2 and 3 (Figure 1A). The second genotype used in this paper is the mutant line *dfa2h* ^*2*^, in which the transposable element is inserted in exon 4 towards the end of the gene (Figure 1A). To exclude potential off-target effects, we generated an additional compound heterozygous mutant line (*dfa2h* ^*1*^ */dfa2h* ^*2*^) by crossing both mutant lines to each other. Human carriers of a heterozygous *FA2H* mutation do not exhibit FAHN-related signs or symptoms; hence we used a heterozygous *dfa2h* control (*control* ^*het*^ *)* in addition to the wild-type control flies (*control* ^*WT*^). To determine the relative dfa2h expression levels in these mutant flies, *dfa2h* cDNA from one-week-old flies was examined by quantitative PCR. As expected, the *control* ^*het*^ revealed a cDNA level of 52% compared to the *control* ^*WT*^. In comparison, the *dfa2h* cDNA in homozygous and compound heterozygous alleles ranged between 1 and 3%, resulting in an almost complete lack of *dfa2h* cDNA (Figure 1B).

FAHN patients exhibit severe disease symptoms early in life, leading to premature death (Gregory et al., 2011). Hence, the lifespan of loss of dfa2h flies was monitored. The 50% survival rate of *control* ^*het*^ flies was not decreased but slightly increased compared to *control* ^*WT*^ flies (Figure 1C). Similar to what is observed in FAHN patients, flies with complete loss of dfa2h showed a substantial reduction in survival probability, and the median survival is reduced by half compared to the control flies (Figure 1C).

### 3.2 Loss of dfa2h induces progressive impairment of locomotion

FAHN is a movement disorder that presents with ataxia, dystonia, and spasticity (Rattay et al., 2019). To test locomotion in flies, groups of four to five flies were screened for movement impairment. One-week-old *dfa2h*-mutant flies displayed a variety of movement disorders, including shaking, uncoordinated flying, and decreased activity (Figure 1D; Supplementary Video S1), whereas both control flies did not show strong movement abnormalities (Figure 1D). The observed movement disabilities progressively worsened in three-week-old dfa2h-deficient flies (Figure 1E). In addition, one-week-old flies showed reduced flying ability following the loss of dfa2h that further declined in three-week-old flies, compared to the *control* flies (Figures 1F, G). In many neurodegenerative *Drosophila* models, wing position is altered, such as in PD models with a *pink1* mutation (Clark et al., 2006; Park et al., 2006; Fernandes and Rao, 2011; Füger et al., 2012). We observed a similar wing phenotype following the loss of dfa2h (Figures 1H, I). In the *dfa2h*-mutant flies, raised wings were observed, although this was a rather rare finding in *dfa2h* ^*2*^-mutant flies (Figure 1I and Supplementary Table S1). However, loss of dfa2h results in various locomotion defects that progressively exacerbate. Interestingly, when comparing the findings in *control* ^*WT*^ and *control* ^*het*^ flies, it appears that the *control* ^*WT*^ flies show stronger phenotypic changes than the *control* ^*het*^, especially upon aging. Altogether, these findings underscore the *control* ^*het*^ flies as the most appropriate control, which is in agreement with the observation that heterozygous mutant carriers in humans are asymptomatic and are not at risk to develop FAHN (Gregory et al., 2011). Hence, the *control* ^*het*^ is used as a control in the following experiments.

### 3.3 Autophagy is affected following loss of FA2H

**FIGURE 2:**
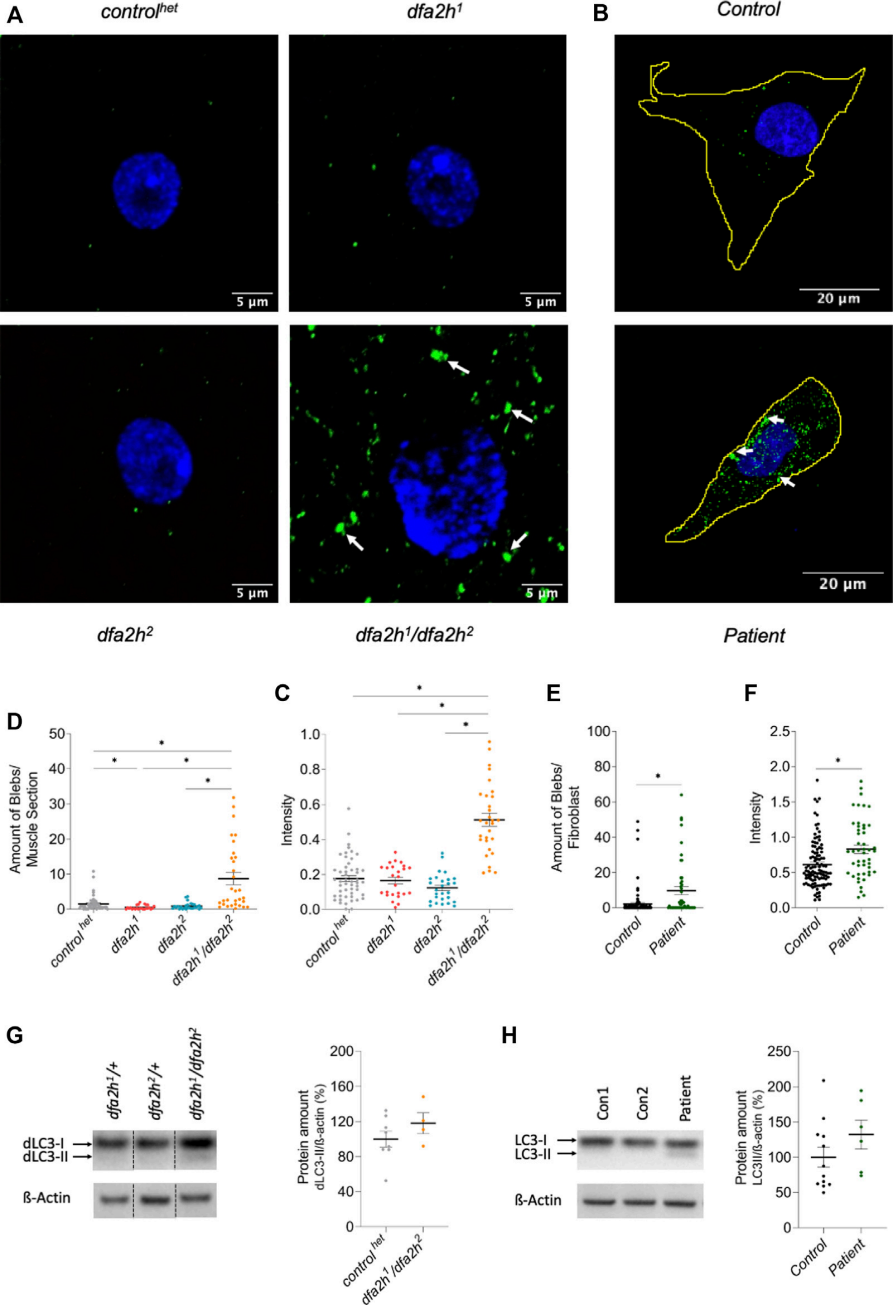
Lack of FA2H leads to altered autophagy. **(A–C)** Immunolabeling of larval muscle sections using autophagy marker dLC3. **(A)** Confocal microscopy images show autophagy alteration in compound heterozygous *dfa2h* mutant. The green labeling shows dLC3 labeling visualizing autophagy, and the blue signal DAPI labeling to visualize the nucleus. The arrows indicate dLC3 accumulations. **(B)** *dfa2h* ^*1*^ */dfa2h* ^*2*^ mutant flies present with a higher number of blebs bigger than 0.1 µm^2^ compared to *control* ^*het*^ and homozygous *dfa2h* mutants. **(C)** Compound heterozygous mutant demonstrates stronger mean intensity of fluorescent signal compared to *control* ^*het*^ and homozygous *dfa2h* mutants. The dots represent single data points, and the lines show mean ± SEM (*n* = 25–50). **(D–F)** Immunolabeling of patient-derived fibroblasts using the autophagy marker LC3. **(D)** Confocal microscopy images demonstrate autophagy alteration upon loss of FA2H. The green fluorescence shows LC3 labeling visualizing autophagy, and the blue signal DAPI labeling to visualize the nucleus. The arrows indicate LC3 accumulations. **(E)** Patient-derived fibroblasts show a higher number of blebs bigger than 0.4 µm^2^. **(F)** Lack of FA2H presents with an increased mean intensity of the fluorescent signal. The dots represent single data points, and the lines show mean ± SEM (n = 48-105). The Kruskal–Wallis test was performed for group comparison with a significance level of *p* < 0.05 (B-C*). For pairwise comparison, Mann-Whitney-U-Test was performed. **(G–H)** Western blot analysis of LC3 expression level. **(G)** Compound heterozygous *dfa2h* ^*1*^ */dfa2h* ^*2*^ flies present with an increase in dLC3-II levels, indicating alterations of autophagy. These findings are consistent with the findings in **(H)** fibroblasts. The expression levels were normalized to ß-Actin expression. The dots represent single data points as percentages, with the controls set to 100%. The lines show mean ± SEM (n_flies_ = 4–8; n_fibroblasts_ = 6–12).

The removal of cellular compartments, termed autophagy, is a common feature that is damaged in neurodegenerative disorders (Nixon, 2013; Menzies et al., 2017). *dfa2h*-mutant larvae were labeled with the autophagy marker dLC3, commonly used to assess autophagy function. The fluorescence pattern exhibited a substantial accumulation of dLC3 in the compound heterozygous *dfa2h* mutant compared to the *control* ^*het*^; however, no effect was observed in the homozygous mutants (Figures 2A, B). In addition, dLC3 intensity was increased in the compound heterozygous allele, while yet again, no difference was observed in the *dfa2h* homozygous alleles (Figures 2A–C). LC3 labeling in patient-derived fibroblasts showed LC3 accumulation and significantly higher intensity than control fibroblasts (Figures 2D–F), similar to what we observed in the compound heterozygous *dfa2h*-mutant flies. This demonstrates that the findings in the compound heterozygous mutant are evolutionarily conserved. Thus, this mutant is the most disease-relevant one and used for the following experiments. To further validate our results, we performed Western blot analyses using a dLC3 antibody and ß-Actin as a loading control. The evaluation revealed an increase in the amount of LC3-II in flies (Figure 2G) and patient-derived fibroblasts (Figure 2H), which is consistent with the immunolabeling results. Thus, loss of FA2H clearly shows alterations in autophagy and this observation is evolutionarily conserved.

### 3.4 FA2H deficiency induces mitochondrial alteration

**FIGURE 3:**
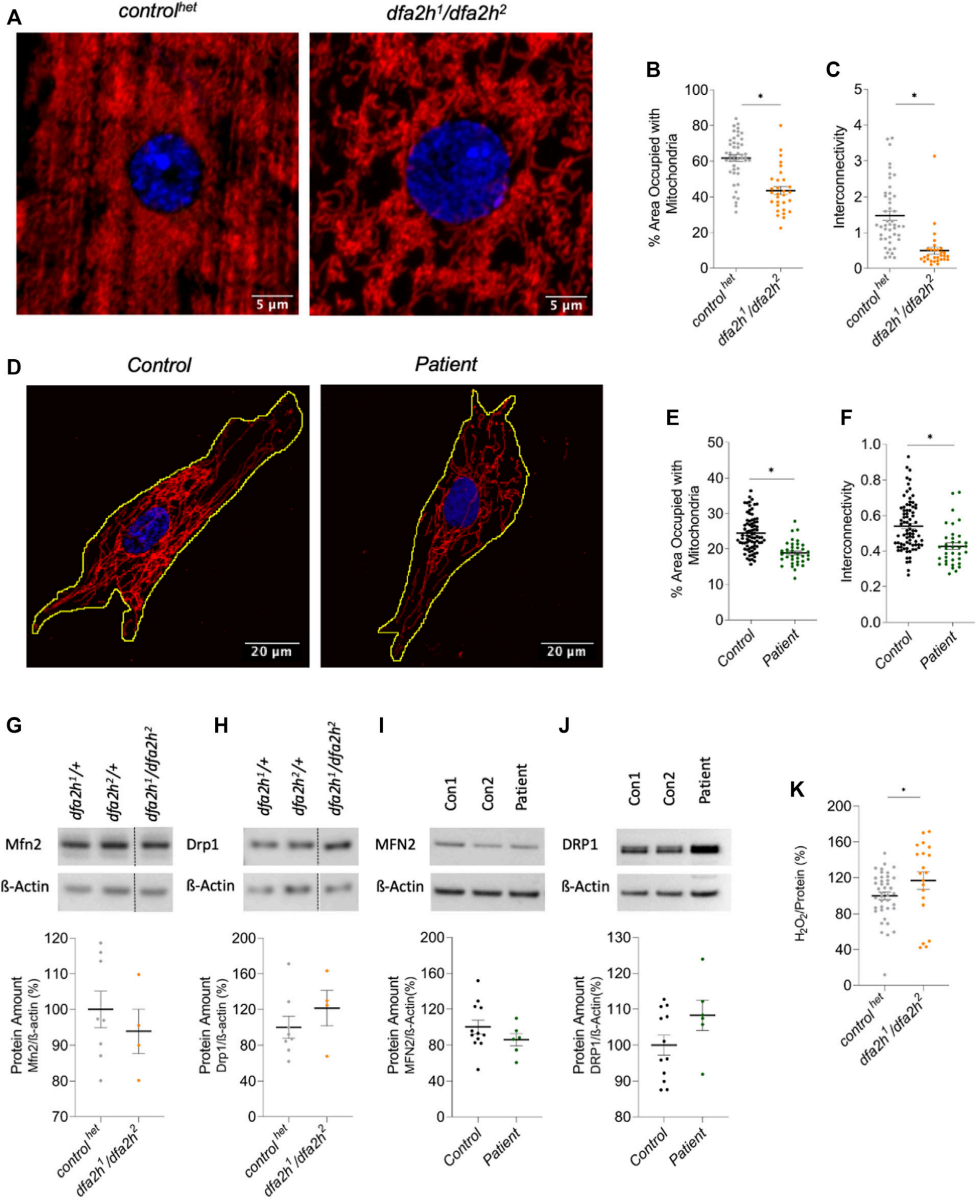
Immunolabeling reveals mitochondrial changes upon loss of FA2H. **(A–C)** Larval muscle sections labeled with mitochondrial marker ATP5a. **(A)** Confocal microscopy images show mitochondrial alteration in loss of *dfa2h* larval muscle sections. The red signal shows ATP5a labeling visualizing mitochondria, and the blue fluorescence DAPI labeling to visualize the nucleus. **(B)** *dfa2h* mutant flies present with less area covered with mitochondria compared to *control* ^*het*^ and **(C)** reduced interconnectivity calculated by area/perimeter ratio. The dots represent single data points, and the lines show mean ± SEM (*n* = 25–46). **(D–F)** Immunolabeling patient-derived fibroblasts using mitochondrial marker GRP75. **(D)** Confocal microscopy images reveal mitochondria alteration. The red signal shows GRP75 labeling visualizing mitochondria, and the blue labeling DAPI labeling to visualize the nucleus. **(E)** Patient-derived fibroblasts present with a lower percentage of area covered with mitochondria and **(F)** reduced interconnectivity calculated by area/perimeter ratio. Dots represent single data points, and the lines show mean ± SEM (*n* = 36–79). For pairwise comparison, Mann-Whitney-U-Test was performed. All pairwise tests, with *p* < 0.05, were indicated with asterisks in the figure. **(G–J)** Western blot analysis to detect alterations of mitochondrial dynamics. **(G)** Compound heterozygous *dfa2h* ^*1*^ */dfa2h* ^*2*^ flies present with decreased Mfn2 expression levels, which is consistent with the findings in **(H)** fibroblasts observing MFN2 levels. **(I)** DRP1 expression levels were increased in loss of dfa2h flies and **(J)** in patient-derived fibroblasts. The expression levels were normalized to ß-Actin expression. The dots represent single data points as percentages, with the controls set to 100%. The lines show mean ± SEM (n_flies_ = 4–8; n_fibroblasts_ = 6–12). **(K)** H_2_O_2_ levels in compound heterozygous *dfa2h* ^*1*^ */dfa2h* ^*2*^ flies are increased compared to *control* ^*het*^ flies. The dots represent single data points as percentages, with the *control* ^*het*^ set to 100%. The lines show mean ± SEM (*n* = 20–40). For pairwise comparison, Mann-Whitney-U-Test was performed. The asterisk in the figure indicates the pairwise test with *p* < 0.05.

In addition to altered autophagy, mitochondrial impairment is a common finding in many neurodegenerative diseases (Carmo et al., 2018; Monzio Compagnoni et al., 2020; Malpartida et al., 2021; Misrani et al., 2021; Vos and Klein, 2021). Hence, we investigated whether loss of dfa2h induces mitochondrial abnormalities in the larval muscle by labeling them with a mitochondrial marker. The following experiments were performed with *control* ^*het*^ flies and the compound heterozygous mutant *dfa2h* ^*1*^ */dfa2h* ^*2*^. Furthermore, patient-derived fibroblasts were analyzed as well. The compound heterozygous mutant flies presented with mitochondria-free areas creating holes in the mitochondrial network in addition to shorter and thicker mitochondrial filaments (Figure 3A). To evaluate these findings, the area of the muscle covered with mitochondria and the mitochondrial interconnectivity were determined *via* an area/perimeter ratio calculation. Both quantitative analyses revealed that dfa2h-deficient flies had reduced mitochondrial coverage and interconnectivity compared to *control* ^*het*^ flies (Figures 3A–C), indicating that impaired mitochondrial morphology is a consequence of dfa2h deficiency. Similar to what we observed in flies, mitochondrial area and interconnectivity were significantly reduced in patient-derived fibroblasts compared to control fibroblasts (Figures 3D–F). Thus, mitochondrial abnormalities following the loss of FA2H are evolutionarily conserved. To further investigate the origin of these alterations in mitochondrial morphology, we analyzed mitochondrial dynamics. We performed Western blot analyses with DRP1 (mitochondrial fission protein) and MFN2 (mitochondrial fusion protein) antibodies. The results in both flies and fibroblasts revealed that the expression of MFN2 (Figures 3G,H) decreased after the loss of FA2H, whereas the expression of DRP1 (Figures 3I,J) increased. MFN2 is responsible for mitochondrial elongation, and DRP1 for fragmentation. Therefore, the results support our hypothesis that loss of FA2H results in a disrupted mitochondrial network and shortened mitochondria caused by dysregulation of mitochondrial fusion and fission. An imbalance in mitochondrial dynamics is often accompanied by changes in reactive oxygen species levels (ROS) (Yu et al., 2008; Twig and Shirihai, 2011; Vásquez-Trincado et al., 2016). Therefore, we performed an H_2_O_2_ analysis to investigate the effects of loss of dfa2h on reactive oxygen species. The assay revealed an increase in H_2_O_2_ levels upon dfa2h deficiency (Figure 3K), suggesting that the dfa2h-dependent mitochondrial defects lead to higher ROS concentrations. In conclusion, our results show that the loss of FA2H causes changes in mitochondria.

### 3.5 Loss of dfa2h can be rescued by overexpression of human FA2H

**FIGURE 4:**
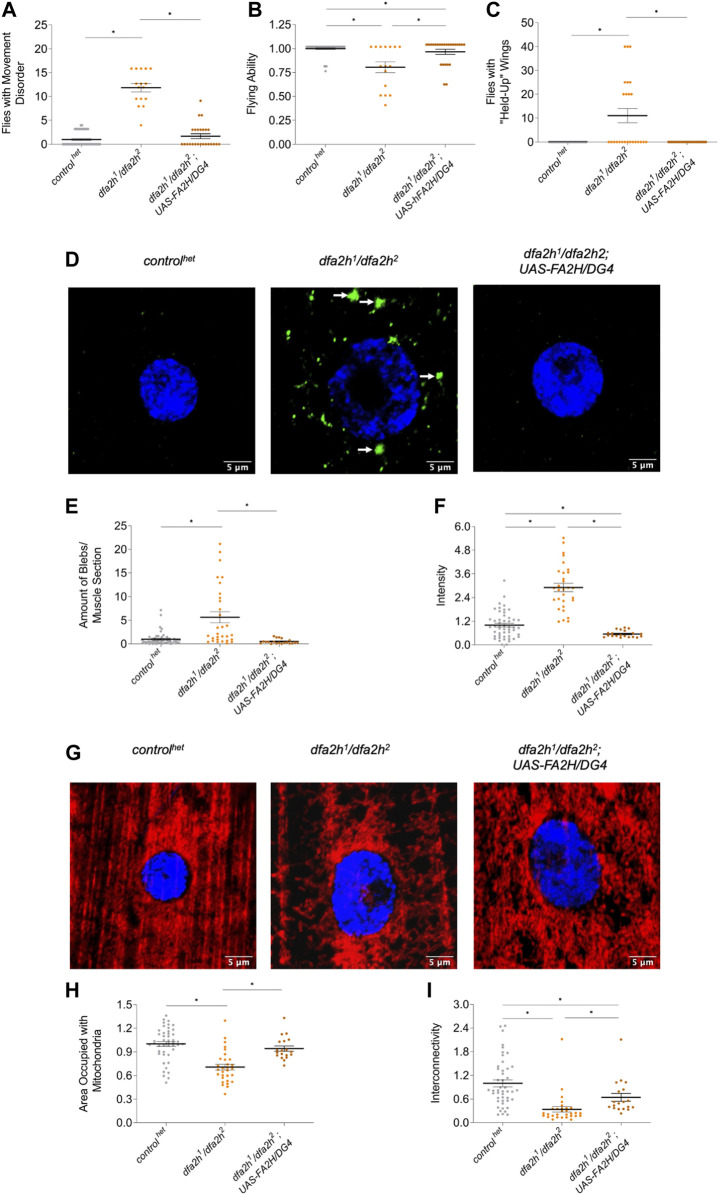
Overexpression of human FA2H rescues the loss of dfa2h phenotypes. The rescue line shows **(A)** reduced number of flies with movement defects, **(B)** increased flight ability, and **(C)** lower number of flies with “held-up” wings compared to the *dfa2h* ^*1*^ */dfa2h* ^*2*^ mutant. For these experiments, groups of four to five flies were formed (n_flight and movement_ = 25–46; n_wing_ = 25–46). **(D)** Immunostaining of larval muscle sections with the autophagy marker dLC3 revealed **(E)** reduced accumulations and **(F)** intensity in rescue flies compared to compound heterozygous mutants (*n* = 20–50). The arrows indicate dLC3 accumulations. The green labeling displays dLC3 labeling to visualize autophagy, whereas the blue signal represents DAPI labeling to visualize the nucleus. **(G)** Larval muscle sections labeled with the mitochondrial marker ATP5a show **(H)** increased mitochondrial density and **(I)** interconnectivity in the rescue line compared with the *dfa2h* ^*1*^ */dfa2h* ^*2*^ mutant line (*n* = 18–46). The red signal shows ATP5a labeling to visualize mitochondria, and the blue signal shows DAPI labeling to visualize the nucleus. The dots in all these graphs represent individual data points. The controls are set to 1, and the lines show the mean ± SEM. The Kruskal–Wallis test was performed for group comparison with a significance level of *p* < 0.05 (A–C, E–F, H–I*).

To test whether the observed effects were indeed a consequence of the loss of dfa2h, we overexpressed human wild type FA2H in fa2h-deficient flies and investigated whether this resulted in partial or complete rescue. Overexpression of human FA2H rescued movement abnormalities (Figure 4A), flight ability (Figure 4B), and wing positioning (Figure 4C) in flies with dfa2h loss. In addition, immunostaining of dLC3 in larval muscle cells (Figure 4D) revealed decreased accumulation (Figure 4E) and intensity (Figure 4F), indicating a complete rescue following the expression of human FA2H in *dfa2h*-mutant flies. Furthermore, mitochondrial density and interconnectivity (Figures 4G–I) were partially rescued. Therefore, our results confirm that FA2H function is evolutionarily conserved and, hence, the analysis of the compound heterozygous *dfa2h* ^*1*^ */dfa2h* ^*2*^ line provides new insights into the underlying mechanisms of FAHN disease and as a model for drug screening.

## 4 Discussion

We here characterized and validated a fly model for FAHN that can be used for loss-of-function analyses and drug screening of dfa2h. FAHN is a rare disease, and the few established FAHN models do not yet provide detailed insights into the consequences of loss of FA2H (Potter et al., 2011; Li et al., 2018; Hardt et al., 2020; Mandik and Vos, 2021). Hence, little is known about its underlying disease mechanism. Furthermore, individual model organisms generally do not reflect the entire disease phenotype (Nagoshi, 2018). Consequently, different model organisms are required to reveal the disease mechanisms entirely. The use of *Drosophila* models of various neurodegenerative diseases already revealed valuable findings, such as essential insights into the pathology of Alzheimer’s disease (AD) (Williams et al., 2000; Wittmann et al., 2001; Shulman and Feany, 2003; Finelli et al., 2004) or the interaction of PINK1 and Parkin in PD (Clark et al., 2006; Park et al., 2006). A fly model for FAHN has not been previously established. Here, we examined three different dfa2h-deficient fly lines focusing on abnormalities related to the signs and symptoms observed in FAHN patients.

All fly lines showed a reduced lifespan, locomotor dysfunction, and reduced flying ability, consistent with findings in fly modeling of other neurodegenerative diseases with locomotion defects (Wu et al., 2009; Kinghorn et al., 2015). Notably, the experiments revealed a worse movement phenotype of the *control* ^*WT*^ compared to *control* ^*het*^, which increased with age. Our observations align with previous findings that *control* ^*WT*^ flies exhibit loss of climbing ability, shortened lifespan, and impaired resistance to stress (Ferreiro et al., 2018). Thus, since heterozygous FA2H mutations do not result in any disease phenotypes in flies or humans, it is a valid control. Furthermore, the abnormal wing position was observed in *dfa2h* ^*1*^ and *dfa2h* ^*1*^ */dfa2h* ^*2*^ but not in *dfa2h* ^*2*^. A possible explanation lies in the fact that these mutant lines are transposable insertions. A side effect of transposable elements are random insertions in the genome that are not identified. A homozygous mutant line, therefore, also has these possible random insertions in homozygous conditions. Hence, possible off-target effects are increased while the compound heterozygous mutant lines still have a healthy gene copy. Thus, the observed differences in the homozygous *dfa2h*-mutants can be explained by these off-target effects that worsen the fitness in addition to the effect upon loss of dfa2h. The analysis of dLC3 in larval muscle cells provides additional evidence supporting the unsuitability of the homozygous mutants to investigate loss of fa2h. Here, the accumulation of dLC3, which is present in patient-derived fibroblasts, was detected in the compound heterozygous alleles but not in the homozygous alleles. Thus, we focused on the compound heterozygous line in further experiments.

Based on the results from the LC3 analyses, we conclude that there is an effect on autophagy leading to higher levels of LC3-II. However, further in-depth analyses are required to determine the exact mechanisms resulting in the observed defects.

Our data on mitochondrial phenotypes revealed problems with mitochondrial morphology that correlated with imbalanced mitochondrial dynamics. Multiple other neurodegenerative diseases, such as PD, AD, and Huntington’s disease, have already been proven to have disturbed mitochondrial dynamics (Wang et al., 2008; Manczak et al., 2011; Zhao et al., 2018; Bonello et al., 2019; Yang et al., 2021). Furthermore, ROS levels were increased upon loss of dfa2h. In *Pink1*-related PD, upregulated ROS levels were linked to an upregulation of mitochondrial elongation (Poole et al., 2008). However, there is evidence that increased fragmentation can lead to elevated ROS levels as well (Yu et al., 2008; Twig and Shirihai, 2011; Vásquez-Trincado et al., 2016). Mitochondrial fission-fusion balance is a sensitive system. Even small changes in either direction can substantially affect cellular processes (Chan, 2006; Poole et al., 2008; Vásquez-Trincado et al., 2016). In PD, mitochondrial fragmentation stimulates autophagic processes, thus linking an imbalanced mitochondrial network to autophagy (Poole et al., 2008; Narendra et al., 2009).

The rescue experiments on behavioral abnormalities and immunolabeling showed that the expression of human FA2H partially or fully restored the phenotypes of dfa2h loss. This provides evidence that the observed phenotypes are indeed caused by the absence of dfa2h and links the function of FA2H in humans and flies, making our fly model disease-relevant.

Analyses in recent years showed that sphingolipid metabolism significantly impacts cellular processes (Alessenko and Albi, 2020; Alaamery et al., 2021; Mandik and Vos, 2021). For instance, amyloid beta accumulation in AD stimulates sphingomyelin hydrolysis resulting in increased ceramide levels in AD patients (Haughey et al., 2010; Filippov et al., 2012; van Echten-Deckert and Walter, 2012). In a loss of Pink1 fly model, we have linked impaired mitochondria to ceramide accumulation (Vos et al., 2021). Hence, ceramide is a key sphingolipid that, upon mitochondrial accumulation, leads to ceramide-induced mitophagy, the autophagy of mitochondria (Sentelle et al., 2012). FA2H synthesizes 2-hydroxysphingolipids and 2-hydroxyglycosphingolipids and is, therefore, part of the sphingolipid metabolism. Further studies are required to analyze the effect of loss of FA2H on the sphingolipid metabolism and, thus, sphingolipid composition and how these alterations modify cellular processes such as autophagy and mitochondrial function that together suggest an effect on the level of mitophagy.

To conclude, we present a new FAHN model that constitutes a loss of dfa2h fly model. Furthermore, we showed mitochondrial dysfunction and altered autophagy to be evolutionarily conserved and relevant in a disease context. Hence, our fly model can be used for drug screening and studies to increase our understanding of the underlying mechanisms enabling the identification of novel therapeutic targets to treat FAHN.

## Data Availability

The raw data supporting the conclusions of this article will be made available by the authors, without undue reservation.

## References

[B1] Alaamery M. Albesher N. Aljawini N. Alsuwailm M. Massadeh S. Wheeler M. A. (2021). Role of sphingolipid metabolism in neurodegeneration. J. Neurochem. 158, 25–35. 10.1111/jnc.15044 32402091 PMC7665988

[B2] Alderson N. L. Rembiesa B. M. Walla M. D. Bielawska A. Bielawski J. Hama H. (2004). The human FA2H gene encodes a fatty acid 2-hydroxylase. J. Biol. Chem. 279, 48562–48568. 10.1074/JBC.M406649200 15337768

[B3] Alessenko A. V. Albi E. (2020). Exploring sphingolipid implications in neurodegeneration. Front. Neurol. 11, 437. 10.3389/fneur.2020.00437 32528400 PMC7254877

[B4] Arber C. E. Li A. Houlden H. Wray S. (2016). Review: Insights into molecular mechanisms of disease in neurodegeneration with brain iron accumulation: Unifying theories. Neuropathol. Appl. Neurobiol. 42, 220–241. 10.1111/nan.12242 25870938 PMC4832581

[B5] Bier E. (2005). Drosophila, the golden bug, emerges as a tool for human genetics. Nat. Rev. Genet. 6, 9–23. 10.1038/nrg1503 15630418

[B6] Bischof J. Maeda R. K. Hediger M. Karch F. Basler K. (2007). An optimized transgenesis system for Drosophila using germ-line-specific phiC31 integrases. Proc. Natl. Acad. Sci. U. S. A. 104, 3312–3317. 10.1073/PNAS.0611511104 17360644 PMC1805588

[B7] Bolus H. Crocker K. Boekhoff-Falk G. Chtarbanova S. (2020). Modeling neurodegenerative disorders in drosophila melanogaster. Int. J. Mol. Sci. 21, 3055. 10.3390/ijms21093055 32357532 PMC7246467

[B8] Bonello F. Hassoun S. M. Mouton-Liger F. Shin Y. S. Muscat A. Tesson C. (2019). LRRK2 impairs PINK1/Parkin-dependent mitophagy via its kinase activity: Pathologic insights into Parkinson’s disease. Hum. Mol. Genet. 28, 1645–1660. 10.1093/HMG/DDZ004 30629163

[B9] Carmo C. Naia L. Lopes C. Rego A. C. (2018). Mitochondrial dysfunction in huntington’s disease. Adv. Exp. Med. Biol. 1049, 59–83. 10.1007/978-3-319-71779-1_3 29427098

[B10] Chan D. C. (2006). Mitochondrial fusion and fission in mammals. Annu. Rev. Cell Dev. Biol. 22, 79–99. 10.1146/ANNUREV.CELLBIO.22.010305.104638 16704336

[B11] Clark I. E. Dodson M. W. Jiang C. Cao J. H. Huh J. R. Seol J. H. (2006). Drosophila pink1 is required for mitochondrial function and interacts genetically with parkin. Nature 441, 1162–1166. 10.1038/nature04779 16672981

[B12] Dagda R. K. Cherra S. J. Kulich S. M. Tandon A. Park D. Chu C. T. (2009). Loss of PINK1 function promotes mitophagy through effects on oxidative stress and mitochondrial fission. J. Biol. Chem. 284, 13843–13855. 10.1074/JBC.M808515200 19279012 PMC2679485

[B13] Darley F. L. Aronson A. E. Brown J. R. (1969). Differential diagnostic patterns of dysarthria. J. Speech Hear. Res. 12, 246–269. 10.1044/jshr.1202.246 5808852

[B14] Eckhardt M. Yaghootfam A. Fewou S. N. Zöller I. Gieselmann V. (2005). A mammalian fatty acid hydroxylase responsible for the formation of alpha-hydroxylated galactosylceramide in myelin. Biochem. J. 388, 245–254. 10.1042/BJ20041451 15658937 PMC1186713

[B15] Edvardson S. Hama H. Shaag A. Gomori J. M. Berger I. Soffer D. (2008). Mutations in the fatty acid 2-hydroxylase gene are associated with leukodystrophy with spastic paraparesis and dystonia. Am. J. Hum. Genet. 83, 643–648. 10.1016/j.ajhg.2008.10.010 19068277 PMC2668027

[B16] Fernandes C. Rao Y. (2011). Genome-wide screen for modifiers of Parkinson’s disease genes in Drosophila. Mol. Brain 4, 17. 10.1186/1756-6606-4-17 21504582 PMC3094290

[B17] Ferreiro M. J. Pérez C. Marchesano M. Ruiz S. Caputi A. Aguilera P. (2018). *Drosophila melanogaster* white mutant w1118 undergo retinal degeneration. Front. Neurosci. 11, 732. 10.3389/fnins.2017.00732 29354028 PMC5758589

[B18] Filippov V. Song M. A. Zhang K. Vinters H. v. Tung S. Kirsch W. M. (2012). Increased ceramide in brains with Alzheimer’s and other neurodegenerative diseases. J. Alzheimers Dis. 29, 537–547. 10.3233/JAD-2011-111202 22258513 PMC3643694

[B19] Finelli A. Kelkar A. Song H. J. Yang H. Konsolaki M. (2004). A model for studying Alzheimer's Abeta42-induced toxicity in *Drosophila melanogaster* . Mol. Cell. Neurosci. 26, 365–375. 10.1016/j.mcn.2004.03.001 15234342

[B20] Füger P. Sreekumar V. Schüle R. Kern J. V. Stanchev D. T. Schneider C. D. (2012). Spastic paraplegia mutation N256S in the neuronal microtubule motor KIF5A disrupts axonal transport in a Drosophila HSP model. PLoS Genet. 8, e1003066. 10.1371/journal.pgen.1003066 23209432 PMC3510046

[B21] Gregory A. Venkateswaran S. Hayflick S. J. (2011). Fatty acid hydroxylase-associated neurodegeneration. *GeneReviews® [Internet]* . 21735565

[B22] Hardt R. Jordans S. Winter D. Gieselmann V. Wang-Eckhardt L. Eckhardt M. (2020). Decreased turnover of the CNS myelin protein Opalin in a mouse model of hereditary spastic paraplegia 35. Hum. Mol. Genet. 29, 3616–3630. 10.1093/hmg/ddaa246 33215680

[B23] Haughey N. J. Bandaru V. V. R. Bae M. Mattson M. P. (2010). Roles for dysfunctional sphingolipid metabolism in Alzheimer’s disease neuropathogenesis. Biochim. Biophys. Acta 1801, 878–886. 10.1016/J.BBALIP.2010.05.003 20452460 PMC2907186

[B24] Julienne H. Buhl E. Leslie D. S. Hodge J. J. L. (2017). Drosophila PINK1 and parkin loss-of-function mutants display a range of non-motor Parkinson’s disease phenotypes. Neurobiol. Dis. 104, 15–23. 10.1016/j.nbd.2017.04.014 28435104 PMC5469398

[B25] Kinghorn K. J. Castillo-Quan J. I. Bartolome F. Angelova P. R. Li L. Pope S. (2015). Loss of PLA2G6 leads to elevated mitochondrial lipid peroxidation and mitochondrial dysfunction. Brain 138, 1801–1816. 10.1093/brain/awv132 26001724 PMC4559908

[B26] Levi S. Tiranti V. (2019). Neurodegeneration with brain iron accumulation disorders: Valuable models aimed at understanding the pathogenesis of Iron deposition. Pharmaceuticals 12, 27. 10.3390/ph12010027 30744104 PMC6469182

[B27] Li Y. Wang C. Huang Y. Fu R. Zheng H. Zhu Y. (2018). C. Elegans fatty acid two-hydroxylase regulates intestinal homeostasis by affecting heptadecenoic acid production. Cell. Physiol. biochem. 49, 947–960. 10.1159/000493226 30184537 PMC6428043

[B28] Malpartida A. B. Williamson M. Narendra D. P. Wade-Martins R. Ryan B. J. (2021). Mitochondrial dysfunction and mitophagy in Parkinson’s disease: From mechanism to therapy. Trends biochem. Sci. 46, 329–343. 10.1016/J.TIBS.2020.11.007 33323315

[B29] Manczak M. Calkins M. J. Reddy P. H. (2011). Impaired mitochondrial dynamics and abnormal interaction of amyloid beta with mitochondrial protein Drp1 in neurons from patients with alzheimer’s disease: Implications for neuronal damage. Hum. Mol. Genet. 20, 2495–2509. 10.1093/HMG/DDR139 21459773 PMC3109997

[B30] Mandik F. Vos M. (2021). Neurodegenerative disorders: Spotlight on sphingolipids. Int. J. Mol. Sci. 22, 11998. 10.3390/ijms222111998 34769423 PMC8584905

[B31] Menzies F. M. Fleming A. Caricasole A. Bento C. F. Andrews S. P. Ashkenazi A. (2017). Autophagy and neurodegeneration: Pathogenic mechanisms and therapeutic opportunities. Neuron 93, 1015–1034. 10.1016/j.neuron.2017.01.022 28279350

[B32] Misrani A. Tabassum S. Yang L. (2021). Mitochondrial dysfunction and oxidative stress in alzheimer’s disease. Front. Aging Neurosci. 13, 57. 10.3389/fnagi.2021.617588 PMC7930231 33679375

[B33] Monzio Compagnoni G. di Fonzo A. Corti S. Comi G. P. Bresolin N. Masliah E. (2020). The role of mitochondria in neurodegenerative diseases: The lesson from alzheimer’s disease and Parkinson’s disease. Mol. Neurobiol. 57 (7), 2959–2980. 10.1007/S12035-020-01926-1 32445085 PMC9047992

[B34] Nagoshi E. (2018). Drosophila models of sporadic Parkinson’s disease. Int. J. Mol. Sci. 19, 3343. 10.3390/ijms19113343 30373150 PMC6275057

[B35] Narendra D. Tanaka A. Suen D. F. Youle R. J. (2009). Parkin-induced mitophagy in the pathogenesis of Parkinson disease. Autophagy 5, 706–708. 10.4161/AUTO.5.5.8505 19377297

[B36] Nixon R. A. (2013). The role of autophagy in neurodegenerative disease. Nat. Med. 19, 983–997. 10.1038/nm.3232 23921753

[B37] Park J. Lee S. B. Lee S. Kim Y. Song S. Kim S. (2006). Mitochondrial dysfunction in Drosophila PINK1 mutants is complemented by parkin. Nature 441, 1157–1161. 10.1038/nature04788 16672980

[B38] Poole A. C. Thomas R. E. Andrews L. A. McBride H. M. Whitworth A. J. Pallanck L. J. (2008). The PINK1/Parkin pathway regulates mitochondrial morphology. Proc. Natl. Acad. Sci. U. S. A. 105, 1638–1643. 10.1073/PNAS.0709336105 18230723 PMC2234197

[B39] Potter K. A. Kern M. J. Fullbright G. Bielawski J. Scherer S. S. Yum S. W. (2011). Central nervous system dysfunction in a mouse model of Fa2H deficiency. Glia 59, 1009–1021. 10.1002/glia.21172 21491498 PMC3094470

[B40] Rattay T. W. Lindig T. Baets J. Smets K. Deconinck T. Söhn A. S. (2019). FAHN/SPG35: A narrow phenotypic spectrum across disease classifications. Brain 142, 1561–1572. 10.1093/brain/awz102 31135052 PMC6536916

[B41] Reiter L. T. Potocki L. Chien S. Gribskov M. Bier E. (2001). A systematic analysis of human disease-associated gene sequences in *Drosophila melanogaster* . Genome Res. 11, 1114–1125. 10.1101/gr.169101 11381037 PMC311089

[B42] Sentelle R. D. Senkal C. E. Jiang W. Ponnusamy S. Gencer S. Panneer Selvam S. (2012). Ceramide targets autophagosomes to mitochondria and induces lethal mitophagy. Nat. Chem. Biol. 8, 831–838. 10.1038/nchembio.1059 22922758 PMC3689583

[B43] Shulman J. M. Feany M. B. (2003). Genetic modifiers of tauopathy in Drosophila. Genetics 165, 1233–1242. 10.1093/genetics/165.3.1233 14668378 PMC1462852

[B44] Twig G. Shirihai O. S. (2011). The interplay between mitochondrial dynamics and mitophagy. Antioxid. Redox Signal. 14, 1939–1951. 10.1089/ARS.2010.3779 21128700 PMC3078508

[B45] Uchida Y. Hama H. Alderson N. L. Douangpanya S. Wang Y. Crumrine D. A. (2007). Fatty acid 2-hydroxylase, encoded by FA2H, accounts for differentiation-associated increase in 2-OH ceramides during keratinocyte differentiation. J. Biol. Chem. 282, 13211–13219. 10.1074/JBC.M611562200 17355976

[B46] van Echten-Deckert G. Walter J. (2012). Sphingolipids: Critical players in Alzheimer’s disease. Prog. Lipid Res. 51, 378–393. 10.1016/j.plipres.2012.07.001 22835784

[B47] Vásquez-Trincado C. García-Carvajal I. Pennanen C. Parra V. Hill J. A. Rothermel B. A. (2016). Mitochondrial dynamics, mitophagy and cardiovascular disease. J. Physiol. 594, 509–525. 10.1113/JP271301 26537557 PMC5341713

[B48] Vos M. Dulovic-Mahlow M. Mandik F. Frese L. Kanana Y. Diaw S. H. (2021). Ceramide accumulation induces mitophagy and impairs β-oxidation in PINK1 deficiency. Proc. Natl. Acad. Sci. U. S. A. 118, e2025347118. 10.1073/PNAS.2025347118/SUPPL_FILE/PNAS.2025347118 34686591 PMC8639384

[B49] Vos M. Klein C. (2021). The importance of drosophila melanogaster research to uncover cellular pathways underlying Parkinson’s disease. Cells 10, 579–622. 10.3390/cells10030579 33800736 PMC7998316

[B50] Wang X. Su B. Siedlak S. L. Moreira P. I. Fujioka H. Wang Y. (2008). Amyloid-beta overproduction causes abnormal mitochondrial dynamics via differential modulation of mitochondrial fission/fusion proteins. Proc. Natl. Acad. Sci. U. S. A. 105, 19318–19323. 10.1073/PNAS.0804871105 19050078 PMC2614759

[B51] Wiemerslage L. Lee D. (2016). Quantification of mitochondrial morphology in neurites of dopaminergic neurons using multiple parameters. J. Neurosci. Methods 262, 56–65. 10.1016/j.jneumeth.2016.01.008 26777473 PMC4775301

[B52] Williams D. W. Tyrer M. Shepherd D. (2000). Tau and tau reporters disrupt central projections of sensory neurons in Drosophila. J. Comp. Neurol. 428, 630–640. 10.1002/1096-9861(20001225)428:4<630::aid-cne4>3.0.co;2-x 11077417

[B53] Wittmann C. W. Wszolek M. F. Shulman J. M. Salvaterra P. M. Lewis J. Hutton M. (2001). Tauopathy in *Drosophila* : Neurodegeneration without neurofibrillary tangles. Science 293, 711–714. 10.1126/science.1062382 11408621

[B54] Wu Z. Li C. Lv S. Zhou B. (2009). Pantothenate kinase-associated neurodegeneration: Insights from a Drosophila model. Hum. Mol. Genet. 18, 3659–3672. 10.1093/hmg/ddp314 19602483

[B55] Yamamoto S. Seto E. S. (2014). Dopamine dynamics and signaling in Drosophila: An overview of genes, drugs and behavioral paradigms. Exp. Anim. 63, 107–119. 10.1538/EXPANIM.63.107 24770636 PMC4160991

[B56] Yang D. Ying J. Wang X. Zhao T. Yoon S. Fang Y. (2021). Mitochondrial dynamics: A key role in neurodegeneration and a potential target for neurodegenerative disease. Front. Neurosci. 15, 359. 10.3389/fnins.2021.654785 PMC8072049 33912006

[B57] Yu T. Sheu S. S. Robotham J. L. Yoon Y. (2008). Mitochondrial fission mediates high glucose-induced cell death through elevated production of reactive oxygen species. Cardiovasc. Res. 79, 341–351. 10.1093/CVR/CVN104 18440987 PMC2646899

[B58] Zhao Y. Sun X. Qi X. (2018). Inhibition of Drp1 hyperactivation reduces neuropathology and behavioral deficits in zQ175 knock-in mouse model of Huntington’s disease. Biochem. Biophys. Res. Commun. 507, 319–323. 10.1016/J.BBRC.2018.11.031 30449600 PMC6299831

[B59] Zöller I. Meixner M. Hartmann D. Büssow H. Meyer R. Gieselmann V. (2008). Absence of 2-hydroxylated sphingolipids is compatible with normal neural development but causes late-onset axon and myelin sheath degeneration. J. Neurosci. 28, 9741–9754. 10.1523/JNEUROSCI.0458-08.2008 18815260 PMC6671223

