# Supplementary figures and images for: A new model for fatty acid hydroxylase-associated neurodegeneration reveals mitochondrial and autophagy abnormalities
Source: Front Cell Dev Biol. 2022 Dec 14;10:1000553. doi: 10.3389/fcell.2022.1000553 (PMC9794614; doi:10.3389/fcell.2022.1000553)

## Slide 1
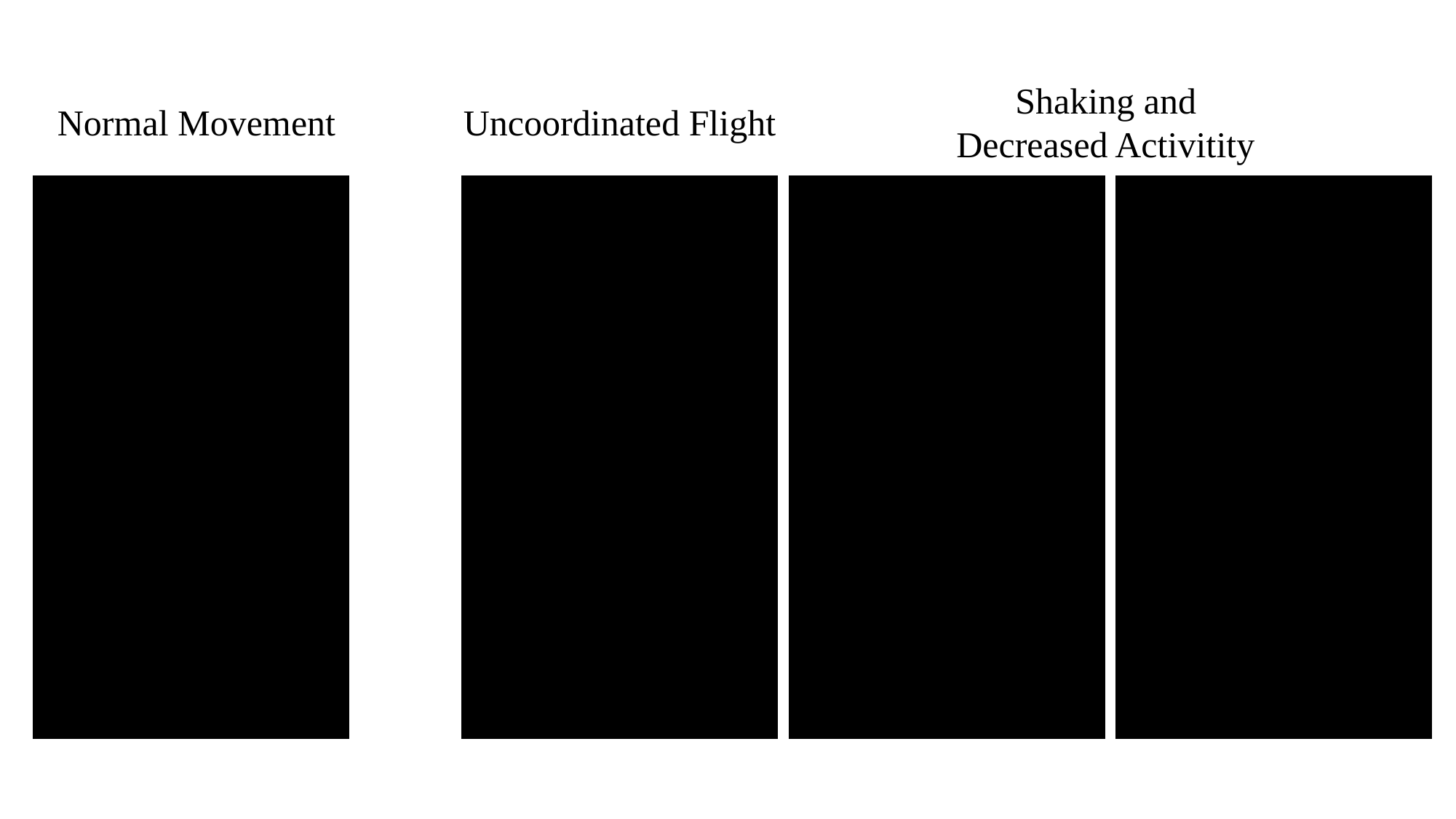

Shaking and
Decreased Activitity
Normal Movement
Uncoordinated Flight

Supplement: Supplementary file 1 [file Presentation1.PPTX]
