# Supplementary material for: A new model for fatty acid hydroxylase-associated neurodegeneration reveals mitochondrial and autophagy abnormalities
Source: Front Cell Dev Biol. 2022 Dec 14;10:1000553. doi: 10.3389/fcell.2022.1000553 (PMC9794614; doi:10.3389/fcell.2022.1000553)
Supplement: Supplementary file 2 [file DataSheet1.docx]

Supplementary Material

**Table S1.** Primer for quantitative PCR. fw: forward primer; rv: reverse primer. The small letter represents the complementary sequence of the vector.

| **Target gene** | **Primer orientation** | **Sequence** | **ID** |
| --- | --- | --- | --- |
| Act5c 1 | fw | TCCACGAGACCACCTACAAC | FBgn0000042 |
|  | rv | CACTTGCGGTGCACAATGGA |  |
| Rpl32 | fw | ATCGGTTACGGATCGAACAA | FBgn0002626 |
|  | rv | GACAATCTCCTTGCGCTTCT |  |
| eEF1α2 | fw | GCGTGGGTTTGTGATCAGTT | FBgn0000557 |
|  | rv | GATCTTCTCCTTGCCCATCC |  |
| dfa2h | fw | TGTGGGATGTCGTCTTCAAA | FBgn0050502 |
|  | rv | GGTATCATTGGAAGTGTATTCCTG |  |
| HiFi | fw | taacagatctgcggccgCAAAATGGCCCCCGCTCC | ENSG00000103089 |
|  | rv | tcacaaagatcctctagaggtaccTCACTGCGTCTTCAGGTGG |  |

#
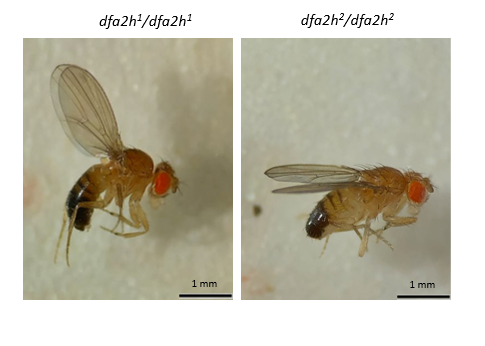


**Figure S1.** Lack of Fa2h exhibits “held-up” wing phenotype in *dfa2h^1^/dfa2h^1^* mutant line but not in *dfa2h^2^/dfa2h^2^*.
